# A microaerobically induced small heat shock protein contributes to *Rhizobium leguminosarum*/*Pisum sativum* symbiosis and interacts with a wide range of bacteroid proteins

**DOI:** 10.1128/aem.01385-24

**Published:** 2024-12-23

**Authors:** Lucía Domingo-Serrano, Claudia Sanchis-López, Carla Alejandre, Joanna Soldek, José Manuel Palacios, Marta Albareda

**Affiliations:** 1 Centro de Biotecnología y Genómica de Plantas, Universidad Politécnica de Madrid - Instituto Nacional de Investigación y Tecnología Agraria y Alimentaria (INIA/CSIC) 16379 , Madrid, Spain; 2 Departamento de Biotecnología-Biología Vegetal, Escuela Técnica Superior de Ingeniería Agronómica, Alimentaria y de Biosistemas, Universidad Politécnica de Madrid 83039 , Madrid, Spain; University of Tennessee at Knoxville, Knoxville, Tennessee, USA

**Keywords:** nitrogen fixation, *Rhizobium*, sHSP, oxidative stress, NCR

## Abstract

During the establishment of the symbiosis with legume plants, rhizobia are exposed to hostile physical and chemical microenvironments to which adaptations are required. Stress response proteins including small heat shock proteins (sHSPs) were previously shown to be differentially regulated in bacteroids induced by *Rhizobium leguminosarum* bv. viciae UPM791 in different hosts. In this work, we undertook a functional analysis of the host-dependent sHSP RLV_1399. A *rlv_1399-*deleted mutant strain was impaired in the symbiotic performance with peas but not with lentil plants. Expression of *rlv_1399* gene was induced under microaerobic conditions in a FnrN-dependent manner consistent with the presence of an anaerobox in its regulatory region. Overexpression of this sHSP improves the viability of bacterial cultures following exposure to hydrogen peroxide and to cationic nodule-specific cysteine-rich (NCR) antimicrobial peptides. Co-purification experiments have identified proteins related to nitrogenase synthesis, stress response, carbon and nitrogen metabolism, and to other relevant cellular functions as potential substrates for RLV_1399 in pea bacteroids. These results, along with the presence of unusually high number of copies of *shsp* genes in rhizobial genomes, indicate that sHSPs might play a relevant role in the adaptation of the bacteria against stress conditions inside their host.

**IMPORTANCE:**

The identification and analysis of the mechanisms involved in host-dependent bacterial stress response is important to develop optimal *Rhizobium*/legume combinations to maximize nitrogen fixation for inoculant development and might have also applications to extend nitrogen fixation to other crops. The data presented in this work indicate that sHSP RLV_1399 is part of the bacterial stress response to face specific stress conditions offered by each legume host. The identification of a wide diversity of sHSP potential targets reveals the potential of this protein to protect essential bacteroid functions. The finding that nitrogenase is the most abundant RLV_1399 substrate suggests that this protein is required to obtain an optimal nitrogen-fixing symbiosis.

## INTRODUCTION

Nitrogen-fixing *Rhizobium*-legume symbiosis is the result of a molecular dialogue established among both partners that leads to the formation of the root nodules containing the symbiosomes, intracellular organelle-like structures in which bacteria are differentiated into bacteroids, the N_2_-fixing form of rhizobia (1, 2). Bacteroids express the nitrogenase enzyme converting N_2_ to ammonia that is secreted to the plant for amino acid synthesis and, in return, the plant provides carbon sources and energy in the form of dicarboxylic acids (2). For the establishment of symbiosis, plant- and bacteria-dependent determinants are required to adjust the behavior of both partners (3, 4). Inside the nodules, bacteroids address a change in their physiology that leads to an adaptation to nitrogen fixation (2–4). Plants provide an adequate ultra-microaerobic environment in the nodule that is required for the synthesis and functioning of the oxygen-sensitive nitrogenase enzyme. Rhizobia sense the low oxygen signal by an adapted regulatory circuit to adjust the expression of *nif* and *fix* genes to this condition (5, 6). Legumes of the Inverted Repeat-Lacking Clade (IRLC) (7), including relevant crops such as alfalfa, vetch, lentil, or pea, produce a family of antimicrobial peptides, called nodule-specific cysteine-rich (NCR) peptides, that induce a terminal bacteroid differentiation (8). The NCR peptides provoke an alteration of membrane permeability, inhibition of cell division, and DNA amplification coupled to cell elongation in vegetative cells, and similar modifications have been observed in bacteroids (9, 10). The cationic NCR peptides have been shown to exhibit antimicrobial activity in vegetative cells leading to a disruption of the integrity of the cell membranes causing a loss of membrane potential (11–14). Bacterial intracellular components have been identified as targets of the NCR peptides in vegetative cells and bacteroids (12), indicating that these peptides have the potential to modulate the bacteroid physiology. The number and the composition of the NCR peptides family vary among IRLC legumes (15), and several bacterial adaptations have been proposed to face exposure to NCR peptides. It has been suggested that these peptides play a role in the microsymbiont discrimination and specificity of the symbiosis (3, 8).

*Rhizobium leguminosarum* bv. viciae (*Rlv*) UPM791 strain is a member of the genospecies E included in the recently described *Rhizobium leguminosarum* species complex (16). *Rlv* effectively nodulates IRLC legume genera belonging to the *Viciae* tribe (*Pisum, Vicia, Lathyrus,* and *Lens*) (17). However, it has been shown that the symbiotic performance is not equally effective in all the combinations, and differences associated with the plant genotype have been reported (18). Likewise, previous studies have shown that the expression of *Rlv* UPM791 hydrogenase, a metalloenzyme that catalyzes the oxidation of H_2_ evolved during the N_2_ fixation, is dependent on the legume host, and permissive (*Pisum*, *Vicia*, and *Lathyrus*) and non-permissive (*Lens*) hosts have been defined (19). A proteomics-based approach identified a relevant number of proteins differentially expressed in bacteroids induced by *Rlv* UPM791 in pea and lentil nodules, among which stress-related proteins, including small heat shock proteins (sHSPs), were identified (20). *Rlv* UPM791 encodes seven sHSPs, one of which was observed to be expressed at higher levels in pea bacteroids (RLV_1399) and three of them were overexpressed in lentil bacteroids (RLV_0502, RLV_0817, and RLV_0818) (20). All the host-dependent sHSPs identified in *Rlv* UPM791 are encoded in plasmids (21) suggesting that these proteins might be involved in the adaptation and survival of rhizobia to the symbiotic environment offered by the different legume hosts. Stress-response proteins upregulated in bacteroids were previously identified by transcriptomic and proteomic analyses in nodule bacteria, indicating that bacteroids are subjected to specific stress symbiotic conditions (22–24). The bacterial tolerance responses to sources of stress in the symbiotic environment have been postulated to be critical for survival within the host and symbiotic performance (4, 25–27). A key trait inside the nodule is the extremely low oxygen tensions, maintained in the low nanomolar range by high-affinity leghemoglobins (8). Rhizobia adapt to such quasi-anoxic conditions by modifying their electron transport chains through the incorporation of high-affinity terminal oxidases. In the case of *Rlv* UPM791, the response to microaerobic conditions is carried out through the action of FnrN. Rhizobial FnrN proteins are members of the CRP/FNR superfamily transcriptional regulators. These regulators control gene expression in response to low oxygen concentrations by binding to anaerobox operators in response to the redox status of a haem group bound to the protein (6). *Rlv* UPM791 contains two functional copies of FnrN (FnrN1 and FnrN2 [28]) and an autoregulation mechanism for their binding to promoter anaerobox sequences has been previously demonstrated (29).

sHSPs are molecular chaperones that maintain protein homeostasis preventing irreversible aggregation of denatured proteins, thus facilitating the substrate protein to be refolded by ATP-dependent chaperone systems (30, 31). Alternatively, irreversibly damaged proteins are degraded by ATP-dependent proteolytic systems (32–34). Biochemically, sHSPs are characterized by their low molecular mass (12–43 kDa) and by a conserved *α*-crystallin domain (ACD) flanked by highly variable and non-conserved N-terminal (NTE) and C-terminal (CTE) extensions (35, 36). sHSPs are present in the three domains of life (35), with a special relevance in multicellular eukaryotes, accounting for more than 30 sHSPs in plants (37, 38). By contrast, most bacterial genomes encode only 1–2 sHSPs but, interestingly, legume endosymbiotic bacteria harbor multiple versions of these proteins (35, 37, 39), likely reflecting the different stresses associated with changes in their lifestyle. Previous studies showed that *Bradyrhizobium diazoefficiens* sHSPs are divided into two classes according to their primary sequence (39). Class A includes bacterial members closely related to *Escherichia coli* IbpA and IbpB and highly similar along their sequences. Class B comprises proteins more divergent in sequence and phylogenetic origin, and prokaryotic and eukaryotic members fall into this class.

Functional studies of sHSPs in plant-associated bacteria are scarce. A role of an sHSP in cyst desiccation resistance in *Azotobacter vinelandii* has been demonstrated (40), and further works have revealed that these proteins promote *Agrobacterium tumefaciens* virulence through the stabilization of a component of the virulence system (41). In addition, several heat-regulated sHSPs whose expression is under the control of ROSE (repression of heat shock gene expression) elements have been identified in rhizobia (32, 39), but the connection of the regulation of these proteins with the symbiosis had not been identified. Previous studies have identified a significant number of cytosolic proteins involved in diverse cellular functions interacting with sHSPs under heat-stress conditions (32, 39), but the analysis of their interactors in legume bacteria has not been addressed so far. In this work, the role of sHSP RLV_1399 in the optimization of the symbiosis of *Rlv* UPM791 with pea plants has been demonstrated. In addition, the *rlv_1399* gene is transcribed from an FnrN-dependent promoter activated under microaerobic free-living conditions. Overexpression of RLV_1399 improves microaerobic culture tolerance to the presence of hydrogen peroxide or antimicrobial NCR peptides suggesting that this protein might protect rhizobia against adverse nodule conditions. The identification of a wide range of bacteroid proteins as co-eluting with RLV_1399 indicates that sHSPs might be a general binding protein stabilizing denatured proteins under symbiotic conditions. The identification of nitrogenase structural subunits as the main proteins associated with this sHSP suggests a relevant role of RLV_1399 for obtaining optimal levels of nitrogen fixation by the *Rhizobium*/legume symbiosis.

## MATERIALS AND METHODS

### Bacterial strains, plasmids, and growth conditions

Strains and plasmids used in this study are listed in Table 1. *R. leguminosarum* strains were routinely grown at 28°C in yeast mannitol broth (YMB) (42), tryptone-yeast extract (TY) (43), *Rhizobium* minimal (Rmin) medium (44), or universal minimal salt (UMS) medium (45). *E. coli* strains were grown at 37°C in LB medium (46). *E. coli* DH5*α* was used for standard cloning procedures (47) and *E. coli* S17.1 (48) for conjugative plasmid transfer between *E. coli* and *R. leguminosarum*. Antibiotic concentrations used were as follows (µg mL^−1^): ampicillin, 100; kanamycin, 50; gentamicin, 5; tetracycline, 5 (for *R. leguminosarum*) or 10 (for *E. coli*).

**TABLE 1:** Bacterial strains and plasmids used in this work

Strain or plasmid	Relevant genotype or phenotype	Source or reference
*Rhizobium leguminosarum*		
UPM791	128C53 wild type; Str^r^ Nod^+^ Fix^+^ Hup^+^	(49)
DG2	UPM791 *fnrN1 fnrN2*	(28)
UPM1421	UPM791 *rlv_1399*::pK18*mob*	This work
*Escherichia coli*		
DH5*α*	*recA1 endA1 gyrA96 thi hsdR17 supE44 relA1* ∆(*lacZYA-argF*)*U169* (Φ80d*lacZ*∆M15) *deoR phoA*	(47)
S17.1	*thi pro hsdR- hsdM +recA* RP4::2-Tc::Mu-Kan::T7, Spec^r^ Str^r^	(48)
Plasmids		
PCR2.1-TOPO	PCR product cloning vector; Amp^r^, Kan^r^	Invitrogen
pBlueScript-II KS+	PCR product cloning vector; Amp^r^	Stratagene
pK18*mob*	Integrative vector pUC18 derivative; *lacZ* mob, Kan^r^	(50)
pK18mob.1399	pK18*mob* containing a 322 bp region internal to *rlv_1399* gene, Kan^r^	This work
pLMB51	*gusA* reporter gene vector with taurine-dependent promoter, Tet^r^	(51)
pLMB1399_ST_	pLMB51 containing *rlv_1399* gene fused to a Strep-tag II coding sequence in its 3′-end and *rlv_1399* promoter, Tet^r^	This work
pBBR1MCS-5	Broad-host-range vector with a P*lac* promoter, Gen^r^	(52)
pBBR1399_ST_	pBBR1MCS-5 containing *rlv_1399* gene fused to a Strep-tag II coding sequence in its 3′-end and *rlv_1399* promoter, Gen^r^	This work

### Determination of bacterial tolerance to oxidative stress and NCR peptides

To study tolerance to oxidative stress, bacterial cultures were grown aerobically in a UMS medium up to OD_600_*≈*0.6 and diluted in a fresh medium to an initial OD_600_ = 0.1. Cell cultures were incubated in 100-well Honeycomb plates (Growth Curves Ltd., Piscataway, New Jersey, United States) with constant, double orbital shaking in a Bioscreen C Pro device (Growth Curves Ltd., Piscataway, New Jersey, United States) at 28°C and 1% O_2_, with optical density measurement intervals of 30 min for 144 h.

To carry out NCR peptides tolerance assays, four NCR peptides in processed form were chemically synthesized (Proteogenix): two peptides identified in pea nodules (G35 and G39) and two identified in lentil nodules (L36 and L40) and showing the highest levels of expression in these legumes (20). Sequences of the peptides are included in Table S1. The lyophilized peptides were resuspended in DMSO at 28 mg mL^−1^ as stock concentrations and diluted with ultrapure water at the required working concentrations. Bacterial cultures were grown microaerobically (1% O_2_) in UMS medium as described (53) up to OD_600_ = 0.1. Then, microaerobic cultures were diluted in fresh medium to OD_600_ = 0.01, and NCR peptides were added at the final concentrations assayed. The mix was incubated for 2 h at 28°C, fivefold serially diluted in 0.9% NaCl and, for each dilution, 5 µL were spotted on TY plates. Growth was evaluated after 72 h of incubation at 28°C.

### Mutant and plasmid construction

DNA manipulations, including purification, restriction, ligation, agarose gel electrophoresis, PCR amplification, and transformation into *E. coli* cells were carried out by standard methods (54). Primers used for mutant and plasmid constructions are included in Table S1.

A mutant derivative bearing a deletion in the *rlv_1399* gene was generated in *Rlv* UPM791 using the pK18*mob* suicide vector (50). For this purpose, a DNA region internal to *rlv_1399* was PCR-amplified using primers rlv_1399_F/rlv_1399_R and genomic DNA from UPM791 as a template. The resulting DNA fragment was cloned in PCR 2.1.-TOPO vector, sequenced, and moved into pK18*mob* vector as a BamHI-XbaI restriction fragment. The resulting plasmid, pK18mob.1399, was transferred into *Rlv* UPM791 by conjugation, and single-crossover-positive colonies were selected by plating on the Rmin medium supplemented with kanamycin. Insertion was verified by PCR using appropriate primers, and the transconjugant strain carrying the mutation in *rlv_1399* was named UPM1421.

For the construction of pLMB1399_ST_ and pBBR1399_ST_ plasmids, the *rlv_1399* gene, including its promoter region, was PCR amplified from *Rlv* UPM791 genomic DNA using primers PromC_rlv_1399_51_BamHI_F/rlv_1399_51_XbaI_Strep_R and Prom_rlv_1399_KpnI_F/ rlv_1399_51_XbaI_Strep_R, respectively. The reverse primers included the sequence coding for *Strep*Tag II peptide (WSHPQFEK) for in-frame fusion of the tag sequence to the 3′-end of the gene. PCR products were cloned separately into pBluescript-II KS+ plasmid using BamHI-XbaI or KpnI-XbaI sites and sequenced. Then, the BamHI-XbaI fragment was cloned upstream of the promoterless *gusA* reporter gene in plasmid pLMB51 (51) rendering pLM1399_ST_ plasmid. The KpnI-XbaI restriction fragment was ligated into the broad host range vector pBBR1MCS-5 (52) resulting in plasmid pBBR1399_ST_.

### Plant assays

Pea (*Pisum sativum* L. cv. Frisson) and lentil (*Lens culinaris* cv. Magda) seeds were surface-sterilized, pregerminated, and seedlings were planted in sterilized Leonard jar-type assemblies with vermiculite as substrate and inoculated with 1 mL of early stationary phase bacterial cultures as previously described (55). Plants were grown in a greenhouse under bacteriologically controlled conditions with a nitrogen-free plant nutrient solution (56) at 18/25°C (night/day) temperatures and 16/8 h of light/dark photoperiod. 21 and 28 days post-inoculation of pea and lentil plants, respectively, shoots were collected and dried (60°C for 48 h), and shoot dry weight was determined. Shoot total nitrogen content was measured using a TruMac C/N analyzer (Leco Corporation). Nitrogenase activity in nodulated roots was determined by the acetylene reduction assay (57).

### RNA extraction and quantitative real-time RT-PCR

RNA extraction from nodule samples and cDNA synthesis were carried out as previously described (58). RNA concentration was quantified with a Nanodrop spectrophotometer and tested for possible DNA contamination by PCR using the RNA samples as templates and primers specific for *rpoD* gen (Table S1). RNA integrity was confirmed by electrophoresis in a 1% agarose gel. Quantitative real-time reverse transcription polymerase chain reactions (qRT-PCR) were performed with the Power SYBR Green master mix (Applied Biosystems) and primers rlv_1399_qPCR_F/rlv_1399_qPCR_R for *rlv_1399,* rlv_0502_qPCR_F/rlv_0502_qPCR_R for *rlv_0502,* rlv_0817_qPCR_F/rlv_0817_qPCR_R for *rlv_0817,* rlv_0818_qPCR_F/rlv_0818_qPCR_R for *rlv_0818,* hupL_qPCR_F/hupL_qPCR_R for *hupL,* and rpoD_qPCR_F/rpoD_qPCR_R for *rpoD* genes (Table S1).

### *β*-Glucuronidase activity assays

*β*-Glucuronidase activity assays were performed as described (59) using X-GlcA (5-bromo 4- chloro-3-indolyl-*β*-D-glucuronide) as substrate, and values of activity were expressed as OD_420_ min^−1^ (mg protein)^−1^. Protein contents of vegetative cell cultures were determined following the bicinchoninic acid method (60) after alkaline digestion of cells at 90°C in 2 N NaOH for 10 min using bovine serum albumin as the standard.

### Purification of RLV_1399-StrepTag II fusion protein

Protein purification was carried out from 5 g of pea nodules. Bacteroids were suspended in 4 mL of buffer W (100 mM Tris–HCl, pH 8, 150 mM NaCl) containing a protease inhibitor mixture (Complete-mini; Roche Diagnostics GmbH), and membrane-free soluble extracts were obtained as previously described (53). Bacteroids were disrupted by three passages through a French pressure cell (SLM Aminco, Silver Spring, MD) at 100 MPa, and soluble fractions were cleared of cell debris and membranes by ultracentrifugation at 135,000 × *g* at 4°C for 1 h. The soluble extract was added to a 0.2 mL *Strep*Tactin Superflow column (IBA, Göttingen, Germany) operated by gravity flow. The column was washed 30 times with 0.2 mL of buffer W to remove unbound proteins, and the tagged protein was eluted by the addition of 600 µL (6 × 100 µL) of buffer W supplemented with 2.5 mM D-desthiobiotin. Relevant eluted fractions were collected, pooled, and concentrated using a centrifugal filter device (Amicon Ultra 0.5 mL, 3K).

### Protein gel electrophoresis and western immunoblot

Proteins were resolved by standard sodium dodecyl sulfate-polyacrylamide gel electrophoresis (SDS-PAGE) and visualized in gels by Coomassie brilliant blue G-250 staining or by immunological detection by western blot assays as previously described (56). NifDK and NifH proteins were detected immunologically using antiserum raised against *A. vinelandii* NifDK (1:8,000 dilution) and NifH (1:4,000 dilution), both a gift from Dr. L. Rubio. Blots were developed using a secondary goat anti-rabbit immunoglobulin G-alkaline phosphatase conjugate (1:3,000 dilution) and a chromogenic substrate (bromochloroindolyl phosphate-nitro blue tetrazolium) as recommended by the manufacturer (Bio-Rad Laboratories, Inc. Hercules, CA, USA). For identification of Strep-tag fusion RLV_1399 protein, *Strep*Tactin conjugated to alkaline phosphatase (1:2,500; IBA, Göttingen, Germany) was used. The images of the Coomassie-stained gels and western blots were analyzed using the ImageJ software (61).

### Protein extract in-gel digestion and proteomic analysis

Protein extracts were subjected to in-gel tryptic digestion and desalted as previously described (62). The desalted protein digests were dried, resuspended in 10 mL of 0.1% formic acid, and analyzed by RP-LC-MS/MS in an Easy-nLC 1200 system coupled to an ion trap LTQ-Orbitrap-Velos-Pro hybrid mass spectrometer (Thermo Scientific) (63). Peptide identification from raw data was carried out using the PEAKS Studio Xpro search engine (Bioinformatics Solutions Inc., Waterloo, Ontario, Canada). Database search was performed against Uniprot-*Rhizobium leguminosarum* bv. viciae UPM791 (7244 entries) + *Pisum sativum* NCRs (52 entries) (decoy-fusion database). The following constraints were used for the searches: tryptic cleavage after Arg and Lys (semi-specific), up to two missed cleavage sites, and tolerances of 20 ppm for precursor ions and 0.6 Da for MS/MS fragment ions, and the searches were performed allowing optional Met oxidation and Cys carbamidomethylation. False discovery rates (FDR) for peptide spectrum matches (PSM) and protein were limited to 0.01. Only those proteins with at least two unique peptides being discovered from LC/MS/MS analyses were considered reliably identified. A similar procedure was used for the identification of specific proteins in excised bands obtained from gels.

Estimation of protein abundance was carried out through a comparison of RPSM values, calculated as RPSM = (PSM/number of predicted tryptic peptides) × 100 (20). The number of predicted tryptic peptides for each protein was calculated using the Expasy PeptideCutter tool (64).

### Phylogenetic analysis

Protein sequences obtained from the GenBank database were aligned using Clustal Omega v.1.2.4 (65). The phylogenetic tree of the protein alignment was inferred using IQ-Tree v.1.6.12 (66) with model testing parameter (-m TEST).

### Statistical analysis

Statistical analysis was performed using an analysis of variance (ANOVA) linear model test, following a completely random design. Multiple comparisons of means were analyzed by Fisher’s protected least significant difference method. The analysis was performed using Statgraphics 19 software.

## RESULTS

### Phylogenetic analysis of rhizobial sHSPs

*Rlv* UPM791 genome encodes 7 sHSPs, five of which are encoded in plasmids (RLV_0361, RLV_0502, RLV_0817, RLV_0818, and RLV_1399) and two in the chromosome (RLV_2751 and RLV_6296) (21). To study the potential diversification in their functional role that might account for the high number of sHSPs, a sequence alignment of sHSPs from *Rlv* UPM791 and other rhizobia was performed, and a phylogenetic tree was generated. In this analysis, the four sHSPs encoded in the genome of *Rhizobium johnstonii* 3841 strain, together with the six sHSPs from *R. leguminosarum* bv. trifolii WSM1325, and the five sHSPs encoded in *Rhizobium etli* CFN42 and *Ensifer meliloti* Rm1021, respectively, were included. In addition, 12 sHSPs from *B. diazoefficiens* USDA110 strain were also considered in the analysis as these proteins have been previously studied to a large extent (32, 39, 67).

**Fig 1:**
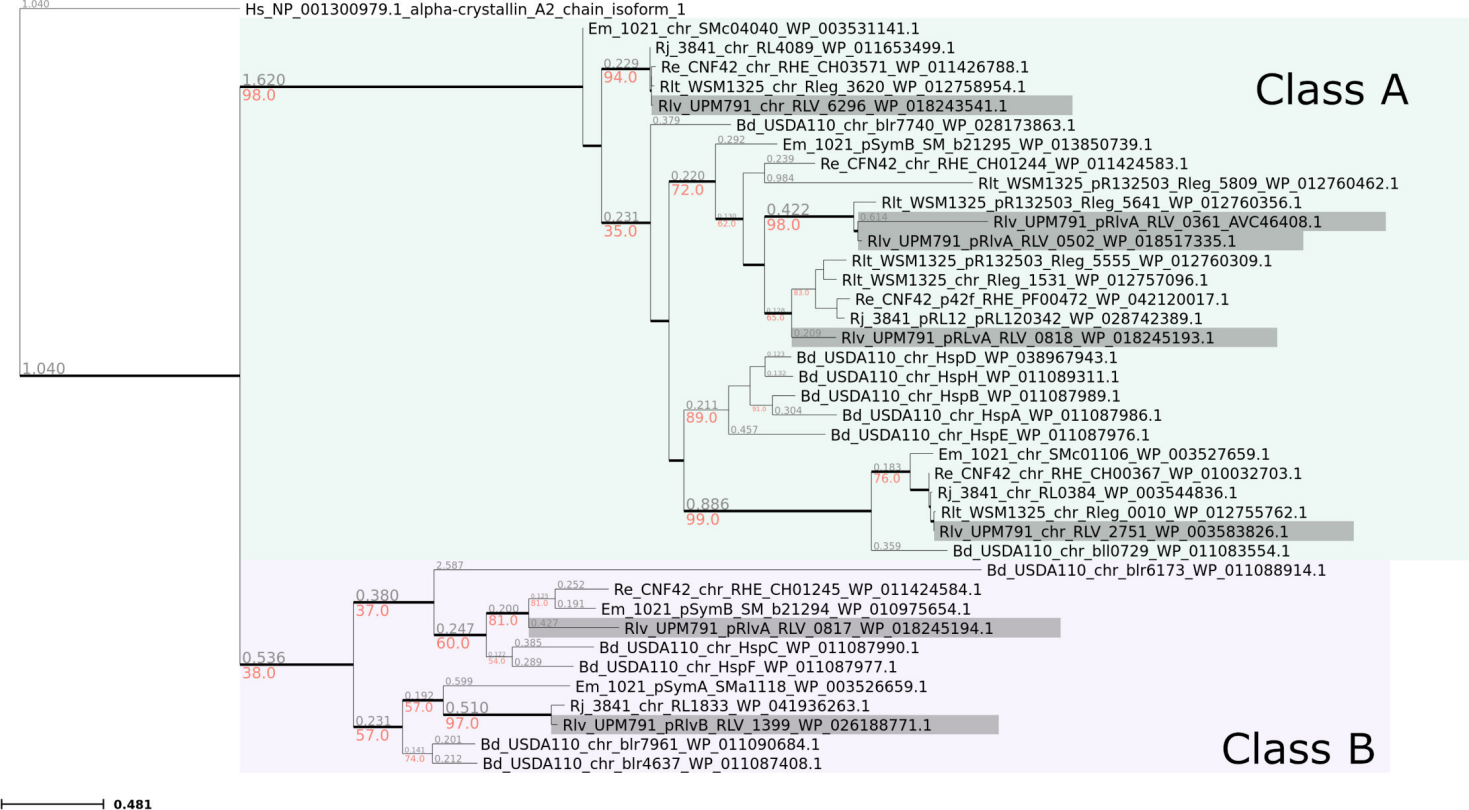
Phylogenetic tree of rhizobial sHSPs. The phylogenetic tree of sequences from rhizobial sHSPs was performed using IQ-Tree from Clustal Omega protein alignment. The alpha-crystallin A2 chain isoform 1 from the human protein CRYAA (NP_001300979_1) was used as a root of the tree. *Rlv* UPM791 sHSPs are highlighted in gray. Two main groups containing Class A (light green) and Class B (light gray) sHSPs are indicated. Numbers in red indicate the bootstrapping support value and numbers in gray indicate the phylogenetic distance. Sequence names shown in the tree contain the abbreviated name of the rhizobial species and strains followed by the name of the sHSP protein, indicating whether the localization of the coding sequence is chromosomic (chr) or plasmidic (p), and their accession numbers from GenBank. Bd: *Bradyrhizobium diazoefficiens*; Em: *Ensifer meliloti*; Re: *Rhizobium etli*; Rj: *Rhizobium johnstonii*; Rlp: *R. leguminosarum* bv. phaseoli; Rlt: *R. leguminosarum* bv. trifolii; Rlv: *R. leguminosarum* bv. viciae.

The phylogenetic tree has shown two clearly differentiated branches. One branch includes Class A proteins previously described in *B. diazoefficiens* HspA, HspB, HspD, HspE, and HspH (39) together with *Rlv* UPM791 RLV_2751, RLV_6296, RLV_0361, RLV_0502, and RLV_0818 proteins (Fig. 1). On the other hand, RLV_1399 and RLV_0817 were clustered together with *B. diazoefficiens* HspC and HspF, which were classified as Class B proteins (39). Most of the rhizobial sHSPs analyzed grouped with *B. diazoefficiens* Class A proteins which are more conserved, with an average percentage of amino acid identity of 76.9 ± 3.6, bearing shorter N-terminal extensions (31 ± 3 amino acids) (Fig. S1). Class B includes proteins that are more divergent in length and sequence (sequence identity 33.2% ± 5.3%) with longer N-terminal extensions (53 ± 3 amino acids). This branch comprises RLV_1399 and RL1883 (its homolog in *R. johnstonii* 3841), one *R. etli* sHSP encoded in the chromosome, and two *E. meliloti* plasmid-encoded sHSPs (Fig. 1). *B. diazoefficiens* also contains three additional sHSPs that, together with HspC and HspF, belong to this class. The phylogenetic tree also shows that some sHSPs from different rhizobial species are clustered together on specific branches and correspond to those encoded in the chromosome. In particular, RLV_2751 and RLV_6296 form two distinctive groups that include the corresponding homologs of the *Rhizobium* species analyzed suggesting that these proteins are highly conserved. Together, these data indicate that *Rlv* UPM791 sHSPs are spread in different branches that might reflect their potential differential function. In addition, sequence comparison suggests sHSPs duplication and diversification events from an ancestor gene.

### RLV_1399 contributes to symbiotic performance in pea plants

The host-dependent sHSP RLV_1399 was shown to be an abundant protein in pea bacteroids, with protein expression levels twofold higher in peas than in lentil bacteroids (20). In addition, the expression of the gene encoding RL1883, the homolog to RLV_1399 in *R. johnstonii* 3841, was highly induced in pea bacteroids (>400-fold) in comparison with the levels of expression in free-living cultures (24). These observations led us to study the potential role of the host-specific RLV_1399 in the symbiosis of *Rlv* UPM791 with pea and lentil plants. Inoculation of pea plants with UPM791 derivative *rlv_1399*-deleted mutant (UPM1421 strain) resulted in a significant decrease on shoot dry weight and nitrogen accumulation values in comparison with those observed with the wild-type strain in this legume (Table 2). On the contrary, the symbiotic performance of the wild-type and mutant strains was statistically similar in lentil plants. The impaired phenotype of *Rlv* UPM1421 in pea was complemented by the expression of wild-type copies of RLV_1399 in pBBR1399_ST_ plasmid. It has to be noted that the introduction of extra copies of the *rlv_1399* gene into the wild-type strain did not result in an improvement of the symbiotic performance in either legume host, indicating that the levels of the bacterial sHSP in pea and lentil bacteroids are not limiting for optimal symbiotic role. Furthermore, the expression of RLV_1399 from pBBR1399_ST_ plasmid in wild-type strain resulted in a reduction of its symbiotic performance in pea plants, suggesting that there may be a dosage effect of Rlv1399 expression, with excess expression being detrimental. Altogether, the data indicate a role of the pea-specific sHSP in the symbiotic performance of *Rlv* UPM791 with pea plants.

**TABLE 2:** Effect of sHSP RLV_1399 on the symbiotic performance of *Rlv* UPM791 with pea and lentil as host plants^*a*^

Strain	Pea		Lentil	
	Shoot dry weight	Shoot nitrogen	Shoot dry weight	Shoot nitrogen
Control	114.6 ± 4.2 c	2.3 ± 0.3 c	102.0 ± 7.6 b	1.8 ± 0.1 b
UPM791	262.6 ± 19.7 a	12.7 ± 1.2 a	186.3 ± 25.0 a	7.1 ± 0.9 a
UPM1421	193.3 ± 7.8 b	9.2 ± 0.5 b	171.1 ± 6.9 a	6.7 ± 0.2 a
UPM791(pBBR1399ST)	235.0 ± 34.8 ab	11.2 ± 0.9 ab	179.4 ± 7.9 a	7.1 ± 0.2 a
UPM1421(pBBR1399ST)	282.1 ± 34.8 a	13.0 ± 1.8 a	193.9 ± 13.1 a	7.6 ± 0.5 a

^*a*^ Data, expressed in mg (plant)^−1^, are means of four replicates ± standard error. Control: uninoculated and non-fertilized plants. Values followed by the same letter, within each column, are not significantly different at *P* < 0.05.

To elucidate whether the defective phenotype observed in pea plants inoculated with the *Rlv* UPM1421 strain had an effect on the nitrogen fixation process, the levels of nitrogenase activity were determined in pea and lentil nodules induced by wild-type and mutant strains. In this experiment, the levels of nitrogenase activity in pea nodules induced by the *rlv_1399* mutant were significantly lower than those observed in the wild-type strain (1559.4 ± 70.4 and 1012.2 ± 86.9 nmoles ethylene. h^−1^ (g nodule fresh weight)^−1^ for wild-type and mutant strains, respectively). On the other hand, the levels of nitrogenase activity in lentil nodules were similar for both strains (1570.95 ± 80.1 and 1497.45 ± 12.8 nmoles ethylene. h^−1^ (g nodule fresh weight)^−1^ for wild-type and mutant strains, respectively). These data indicate that the functional role of sHSP RLV_1399 affects the level of nodule nitrogenase activity in the *Rlv* UPM791 symbiosis with pea plants.

### Regulation analysis of *Rlv* UPM791 sHSP expression

**Fig 2:**
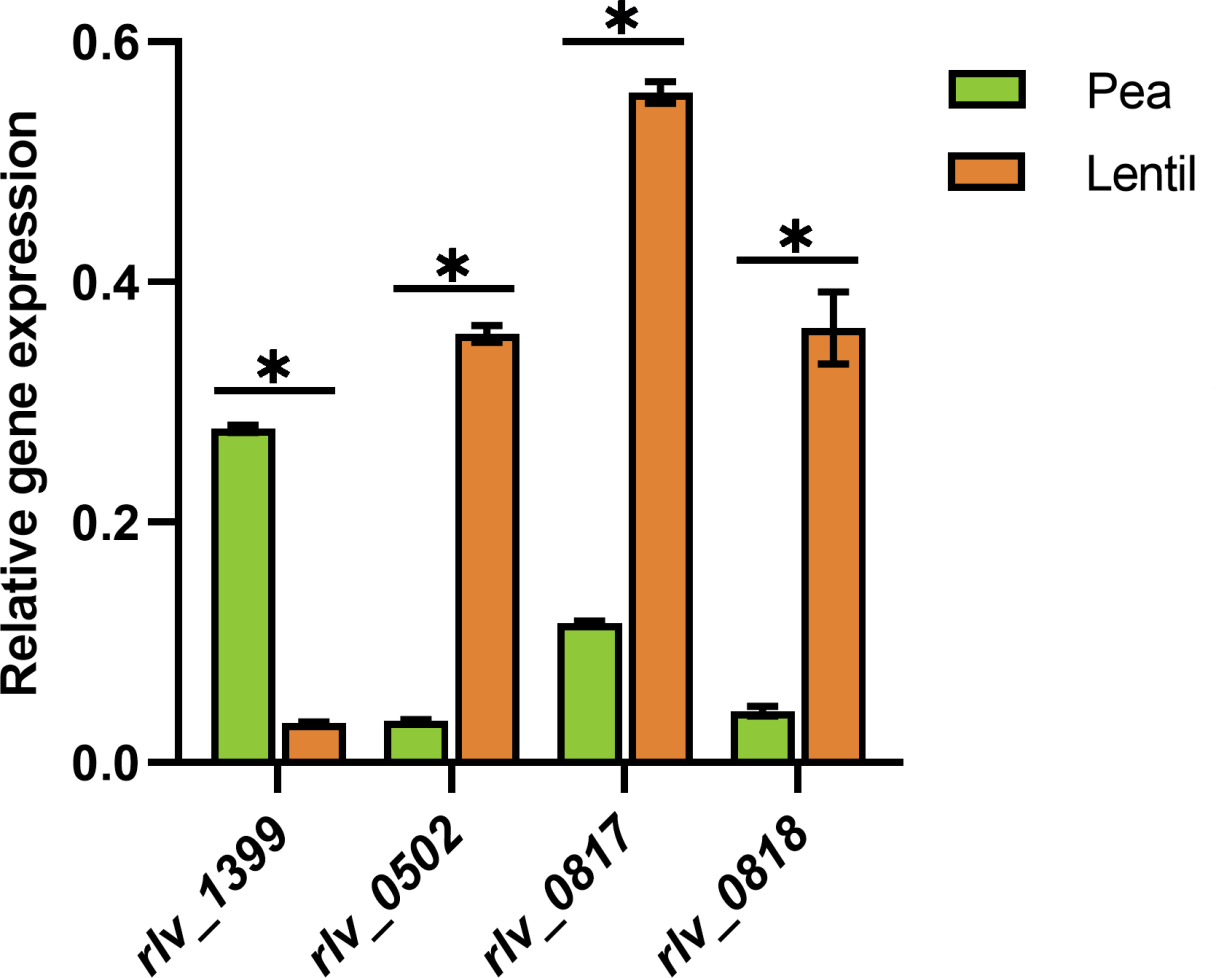
Expression levels of symbiotic sHSP-encoding genes. The histogram shows the relative expression levels of the *shsp* genes in bacteroids induced by *Rlv* UPM791 in pea and lentil nodules using the *rpoD* gene as standard for normalization. Values are the means of three replicates ±standard error. Values significantly different at *P* < 0.01 (*) are indicated.

We first investigated whether the control of the observed host-dependent expression of sHSPs in the *Rlv* UPM791 strain was exerted at the level of transcription. Quantification of the levels of transcripts corresponding to sHSPs previously identified through proteomic analysis (RLV_1399, RLV_0502, RLV_0817, and RLV_0818) revealed that the expression of *rlv_1399* gene was upregulated in pea vs lentil bacteroids, whereas high upregulation in lentil was observed for the three remaining sHSPs (Fig. 2). In these experiments, transcripts of the gene encoding hydrogenase large subunit HupL, known to exhibit a strong host-dependent regulation (19), showed of 80-fold increase in pea vs lentil expression. These results confirm that the expression of sHSPs is dependent on the legume host and is exerted at the transcriptional level.

**Fig 3:**
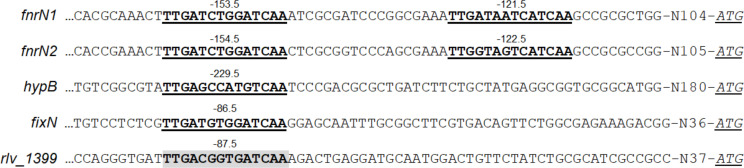
Sequence alignment of anaeroboxes from *Rlv* UPM791 FnrN-type promoters. Shaded box denotes potential FnrN-binding site (consensus anaerobox TTGA-N_6_-TCAA) from DNA upstream of the *rlv_1399* gene. Underlined DNA sequences show anaeroboxes from the promoter regions of *fnrN1*, *fnrN2*, *fixN,* and *hypB* genes. Numbers on the anaeroboxes indicate their centered position to the translation start sites. Promoter sequences were obtained from the corresponding genes in the GenBank database.

To get insight into the regulatory mechanisms governing sHSP expression, we focused our study on the *rlv_1399* gene. Analysis of the sequence of the non-coding region 5′ upstream of the *rlv_1399* gene revealed the existence of a highly conserved anaerobox palindromic sequence (TTGA-n_6_-TCAA) centered at position −87.5 regarding the translation start site of the gene (Fig. 3). A second imperfect anaerobox was also found further upstream in the promoter region at position −143.5 regarding translation start site.

**Fig 4:**
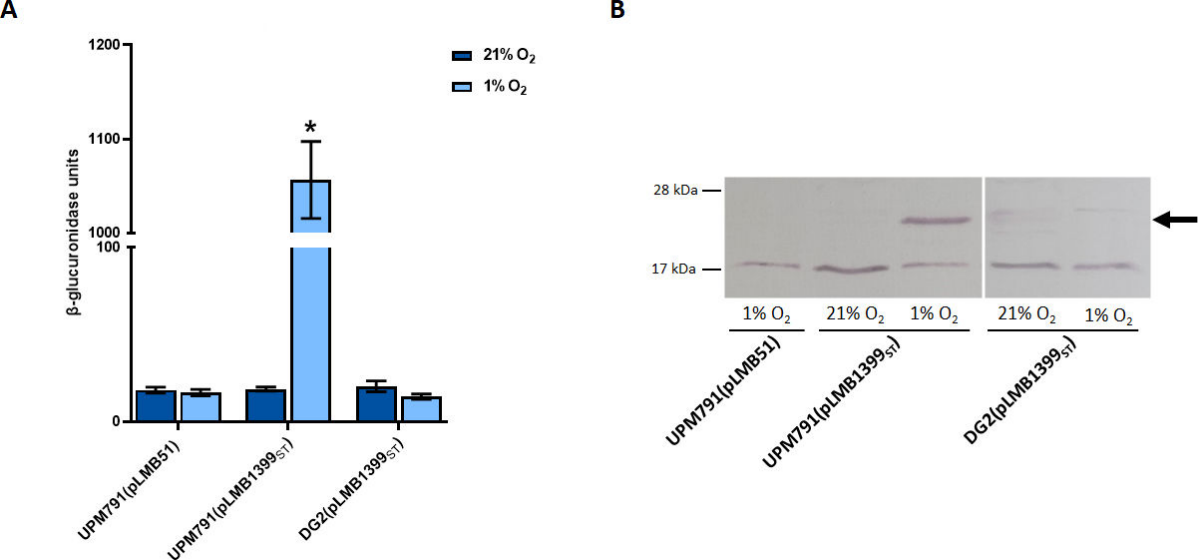
Analysis of the expression of *rlv_1399* in *Rlv* under free-living conditions. (**A**) β-Glucuronidase activity of aerobic (21% O_2_) and microaerobic (1% O_2_) cells. Data are the mean of three replicates ± standard error. Values significantly different at *P* < 0.01 (*) are indicated. (**B**) Immunodetection of RLV_1399_ST_ in cell extracts from bacterial cultures grown under aerobic and microaerobic conditions, as indicated. Proteins were resolved by SDS-PAGE in 15% polyacrylamide gels. Each line was loaded with 90 µg of protein. Numbers on the left margin indicate the position of molecular weight standards (kDa). The arrow on the right indicates the position of RLV_1399_ST_. Left and right panels are portions of the same western blot membrane.

The identification of a potential FnrN-type promoter upstream of the *rlv_1399* gene prompted us to investigate whether *rlv_1399* was microaerobically induced in free-living cells in a FnrN-dependent manner. To carry out this study, a transcriptional fusion of the whole gene and its coding region to a promoterless *gusA* reporter gene was constructed using pLMB51 plasmid. The resulting plasmid, pLMB1399_ST_, also contained a *Strep*Tag sequence at the 3′-end of the cloned gene for monitoring purposes. The plasmid was transferred into UPM791 and DG2 (*fnrN1fnrN2*) strains by conjugation, and the levels of *β*-glucuronidase activity in aerobically and microaerobically grown cells were analyzed. The results showed that levels of reporter activity observed in UPM791(pLMB1399_ST_) grown under microaerobic conditions were *ca*. 50-fold higher than those detected under aerobiosis (Fig. 4A). By contrast, the same fusion induced only basal levels of reporter activities in FnrN-deficient DG2 strain grown under microaerobic conditions. Analysis of crude cell extracts through immunoblot using AP-conjugated *Strep*-Tactin led to the identification of a band with the expected size of sHSP RLV_1399_ST_ in the wild-type microaerobic cells (*ca*. 25 kDa, Fig. 4B). This band was absent in both the DG2 strain grown under the same conditions and in microaerobic cells of UPM791 carrying empty vector included as negative control. In addition, the specific immunoreactive band was not detected when bacterial cultures of the wild-type and DG2 strains were grown under aerobic conditions. Together, these data indicate that the expression of the *rlv_1399* gene is controlled by FnrN in response to low oxygen concentrations under free-living conditions. It has to be noted that an additional, unspecific band of *ca*. 17 kDa was identified in the immunoblots of the crude cell extracts, likely corresponding to a constitutively expressed biotinylated protein. This band was detected in all the strains including UPM791 (pLMB51), negative control, and FnrN-deficient DG2.

### RLV_1399 improves cell tolerance against oxidative stress and NCR antimicrobial peptides

**Fig 5:**
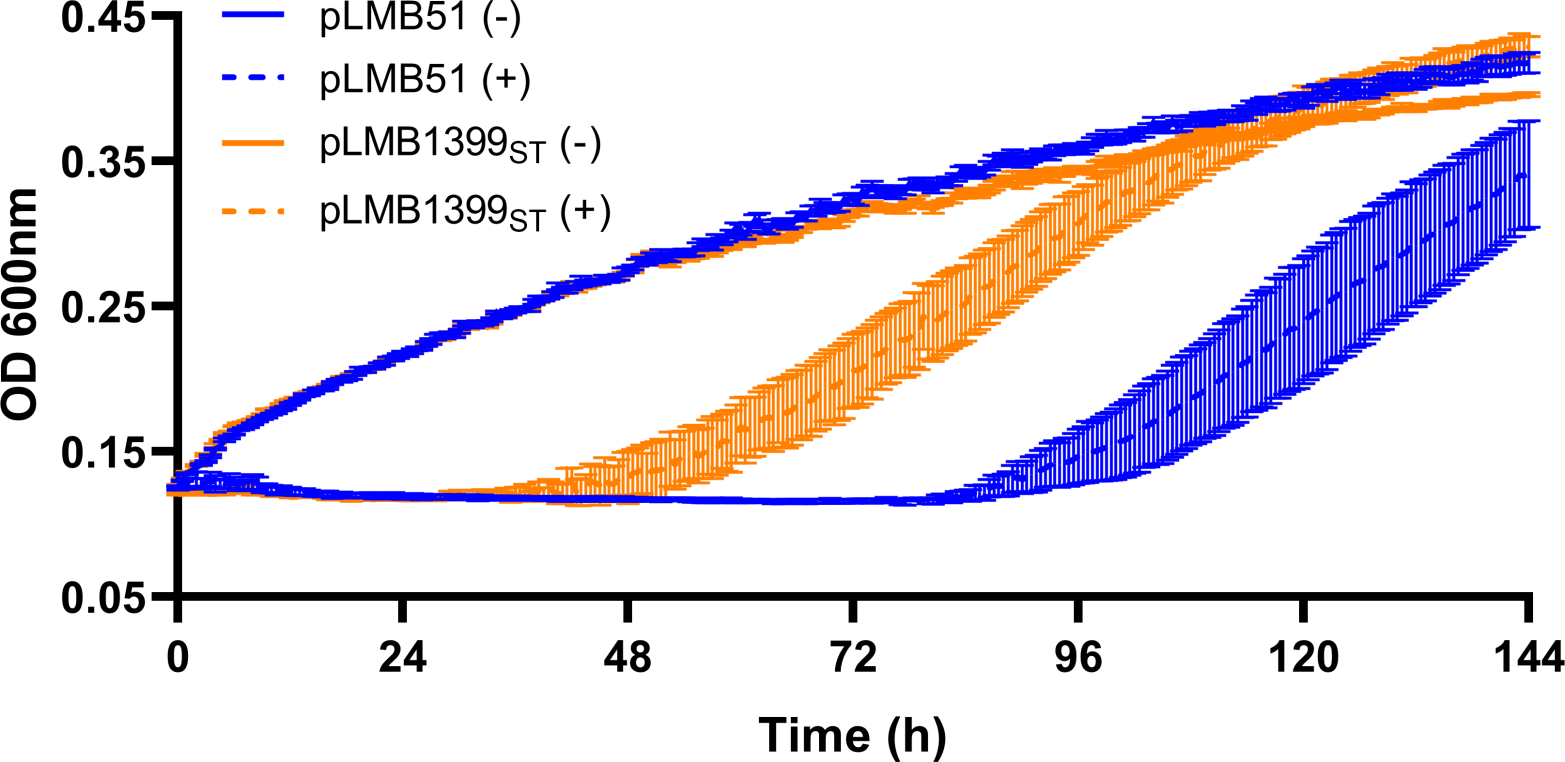
Effect of RLV_1399 expression on the tolerance of *Rlv* to oxidative stress. The graph shows the growth of UPM1421 derivative strains carrying empty vector pLMB51 (blue lines) or pLMB1399_ST_ plasmid (orange lines) grown under microaerobic (1% O_2_) conditions in the absence (−) or presence (+) of 2 mM H_2_O_2_ in UMS medium. Each OD determination represents the mean of three replicates ± standard error.

The microsymbiont is exposed to several stress conditions in the nodule, including the presence of reactive oxygen species produced by both plant and bacteria (68, 69), and NCR antimicrobial peptides in IRLC-legumes (8). To study the effect of RLV_1399 protein on the viability of cells exposed to oxidative stress, the *Rlv* UPM1421 derivative strain harboring pLMB1399_ST_ plasmid, expressing the protein under the control of its promoter, was grown microaerobically in the presence of H_2_O_2_ (2 mM) mimicking the oxidative conditions existing in the nodule. A control strain harboring the empty vector pLMB51 was also included in the analysis. The results indicated that both bacterial strains exhibited similar growth in the absence of the oxidative agent (Fig. 5). The addition of H_2_O_2_ (2 mM) resulted in a significant inhibition of growth in both strains. Interestingly, a higher degree of growth inhibition was observed in the UPM1421(pLMB51) strain exposed to H_2_O_2_, whereas the presence of wild-type copies of *rlv_1399* gene in pLMB1399_ST_ plasmid resulted in partial reversion of the mutant phenotype. A similar growth pattern was observed in the wild-type strain in the absence of the oxidative agent, whereas in the presence of H_2_O_2_, a faster recovery was observed associated with the over-expression of RLV_1399 (Fig. S2). These data indicate that sHSP RLV_1399 improves the growth of microaerobic cells exposed to H_2_O_2_, suggesting that this protein might protect the bacteroids against oxidative stress conditions in the nodule.

**Fig 6:**
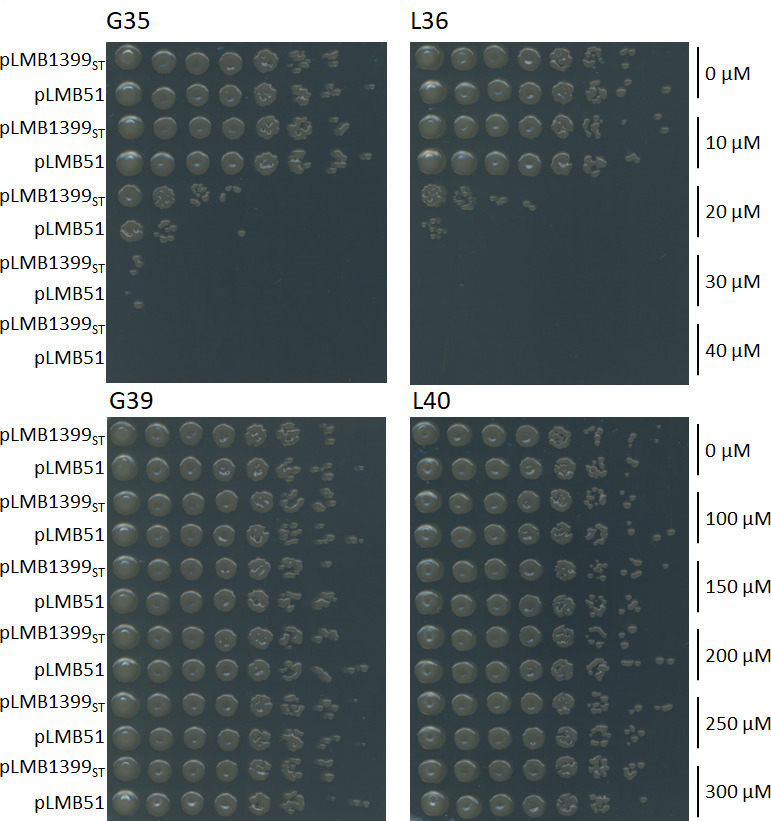
Effect of RLV_1399 expression on cell viability of bacterial cultures exposed to NCR peptides. Microaerobic cells of UPM1421 derivatives strains carrying pLMB51 or pLMB1399_ST_ were exposed to chemically synthesized cationic (**G35, L36**) and anionic (**G39, L40**) NCR peptides from pea (**G**) and lentil (**L**) nodules at the indicated concentrations during 2 h at 28°C. Cells were fivefold serially diluted and 5 µL from each dilution were spotted onto TY plates. The plates were incubated at 28°C for 72 h.

The potential role of the RLV_1399 protein in the protection of bacterial cultures against NCR peptides was also studied. In these analyses, the most abundant anionic and cationic NCR peptides previously identified to exhibit the highest levels of expression in nodules from pea (G35, pI: 9.03; G39, pI: 5.56) and lentil (L36, pI: 8.11; L40, pI: 6.19) plants (20) were tested for their antimicrobial activity. For this purpose, microaerobic (1% O_2_) bacterial cultures were exposed to different concentrations of NCR peptides for 2 h, and cell viability was determined by the drop plate method. The results indicated that the presence of cationic peptides G35 and L36 diminished cell viability in a concentration-dependent manner (Fig. 6). G35 and L36 abolished colony formation in *rlv_1399*-deficient UPM1421(pLMB51) strain at 40 µM and 30 µM, respectively. The expression of wild-type copies of the *rlv_1399* gene in pLMB1399_ST_ plasmid resulted in an improvement of cell viability at 20 µM of both cationic peptides. These results indicate that the cationic NCR peptides exhibit antimicrobial activity against *Rlv* UPM791 and, also, that sHSP RLV_1399 improves the tolerance of bacterial cultures exposed to antimicrobial peptides under these conditions. On the other hand, the anionic peptides G39 and L40 did not show antimicrobial activity against bacterial cultures, even at high peptide concentrations (up to 300 µM, Fig. 6)

### Identification of sHSP RLV_1399-interacting proteins under symbiotic conditions

**Fig 7:**
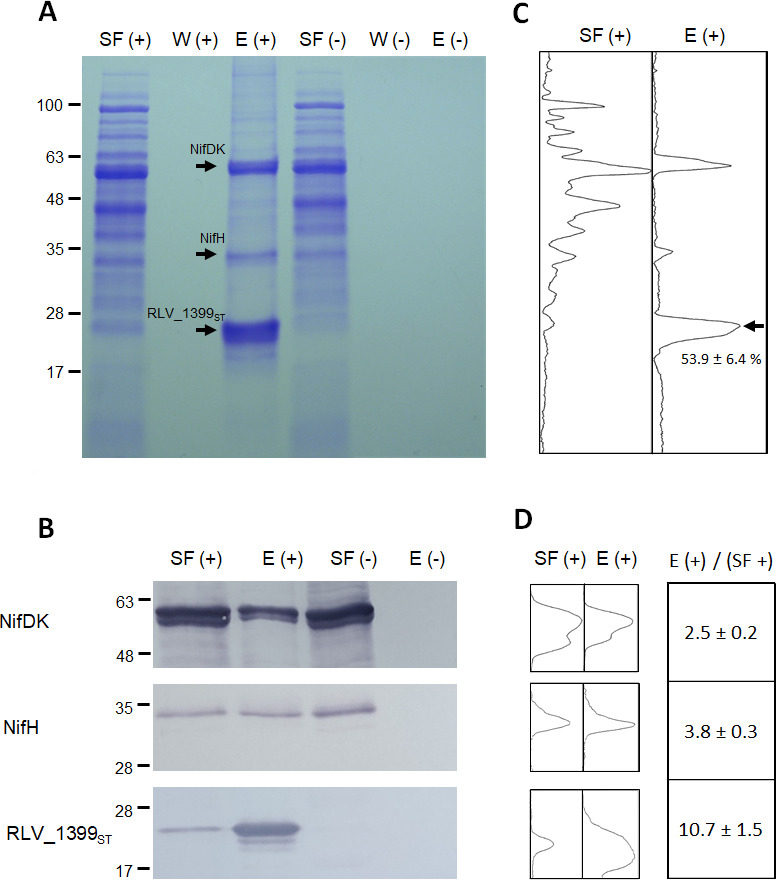
Pull-down assays with sHSP RLV_1399_ST_ protein. Soluble extracts obtained from pea bacteroids induced by UPM1421/pBBR1399_ST_ (+) or UPM1421/pBBR1MCS-5 (−) strains were applied to a *Strep*-Tactin column, and eluted fractions were pooled and concentrated. Proteins were resolved in an 8%–16% polyacrylamide gradient SDS-PAGE gel. (A) Coomassie blue stained gel. The analysis was performed with 10 µg from soluble and pooled eluted fractions. The arrows indicate the bands corresponding to RLV_1399_ST_ and to nitrogenase subunit NifDK and NifH. (B) Immunoblot membranes containing 5 µg of protein from soluble fractions or 2 µg from pooled eluted fractions were revealed using antisera against NifDK, NifH, or with *Strep*Tactin-alkaline phosphatase conjugate to detect RLV_1399_ST_, as indicated. (C) Plots from Coomassie blue stained gels of soluble and eluate fractions from UPM1421(pBBR1399_ST_). The arrow indicates the plot peak corresponding to RLV_1399_ST_. The value shows the mean of the percentage of the RLV_1399_ST_ peak area to the total plot area of three independent stained gels ± standard error. (D) Plots from reactive bands in immunoblots of soluble and eluate fractions from UPM1421(pBBR1399_ST_). Values of increased levels of NifDK, NifH, and RLV_1399_ST_ proteins in the eluate fraction compared to the soluble fraction are indicated. Values are the mean of three independent immunoblot analyses ± standard error. Numbers on the left margins of panels A and B indicate the position of the molecular weight standards (kDa). Plots in panels C and D were generated using Image J software. SF, soluble fraction; W, wash; E, eluate fraction.

To further understand the functional role of RLV_1399 in symbiosis, the potential repertoire of proteins protected by this sHSP in pea bacteroids has been studied. To carry out these analyses, pull-down experiments with soluble extracts from bacteroids induced by UPM1421(pBBR1399_ST_) in pea nodules were performed. Soluble fractions were loaded onto *Strep*Tactin columns, and the pooled desthiobiotin-eluted fractions were concentrated and resolved by SDS-PAGE gels followed by Coomassie blue staining (Fig. 7A). Analysis of the *Strep*-Tactin eluates revealed the presence of multiple bands in the gel. The most prominent band corresponded to the expected size of sHSP RLV_1399_ST_ (*ca*. 25 kDa), which was absent in the eluate from UPM1421(pBBR1MCS-5) included as a negative control. This band was subjected to LC-MS/MS analysis after trypsin digestion. In this analysis, only peptides from RLV_1399 were detected (data not shown).

SDS-PAGE analysis revealed that RLV_1399_ST_ co-purified with multiple potential candidate targets. The eluate was digested with trypsin and subsequently analyzed by LC-MS/MS. The list of 218 proteins consistently identified in the sHSP complexes from two independent replicates is shown in Table S2, where the number of peptide spectrum matches (PSMs) with the *in silico* digested proteome deduced from *Rlv* UPM791 Uniprot database is also included. The 50 most abundant potential sHSP interactors, based on the highest normalized RPSM parameter (see Material and Methods), fell into five main functional categories: nitrogenase-related proteins, stress-related proteins, carbon and nitrogen metabolism, and a variety of proteins involved in essential cellular processes (Table 3).

**TABLE 3:** Major pea bacteroid proteins co-eluting with RLV_1399

Accession	Description	RLV_1399-coeluted proteins		Full bacteroid extracts (20)	
		RPSM^*a*^	Rank^*b*^	RPSM	Rank
Nitrogenase-related proteins					
RLV_1843	Nitrogenase Fe protein (NifH)	2,362	1	200	6
RLV_1842	Nitrogenase protein alpha chain (NifD)	1,522	3	264	5
RLV_1841	Nitrogenase molybdenum-iron protein beta chain (NifK)	1,356	4	379	1
RLV_1892	Protein (FixC)	116	29	54	139
RLV_2767	Scaffold Nfu/NifU N terminal family protein (NifU)	100	32	76	77
RLV_1839	Nitrogenase FeMo cofactor biosynthesis protein (NifN)	72	49	60	84
Stress-related proteins					
RLV_1399	Hsp20/alpha crystallin family protein	2,018	2	146	15
RLV_6296	Hsp20/alpha crystallin family protein	1,181	5	169	93
RLV_4577	Universal stress family protein	456	6	90	54
RLV_3265	60 kDa chaperonin (GroEL)	428	7	285	4
RLV_4730	Universal stress family protein	257	14	96	48
RLV_7164	Chaperone protein (DnaK)	231	16	142	17
RLV_0969	60 kDa chaperonin (GroEL)	165	22	123	32
RLV_0818	Hsp20 family protein	120	27	18	68
RLV_1826	Redoxin family protein	83	37	33	287
RLV_7163	Chaperone protein (DnaJ)	78	40	31	318
RLV_0502	Hsp20/alpha crystallin family protein	75	43	nd	nd
RLV_2751	Hsp20 family protein	75	43	nd	nd
Carbon and nitrogen metabolism-related proteins					
RLV_1846	D-alanine–D-alanine ligase	299	11	297	39
RLV_6165	D-3-phosphoglycerate dehydrogenase	283	13	92	50
RLV_5937	UDP-glucose 6-dehydrogenase	221	19	73	86
RLV_7109	Succinate-semialdehyde dehydrogenase family protein	186	20	130	24
RLV_2756	Methionine adenosyltransferase	159	24	57	129
RLV_2988	UDP-N-acetylglucosamine 1-carboxyvinyltransferase	156	25	17	227
RLV_6528	Aspartokinase	152	26	35	271
RLV_6292	Conserved region in glutamate synthase family protein	89	33	27	368
RLV_1885	Carbamoyl-P synthase L chain ATP-binding domain protein	88	34	69	99
RLV_4559	Pyruvate dehydrogenase E1 component subunit alpha	80	39	45	193
RLV_6681	E2 component of 2-oxoglutarate dehydrogenase complex	76	42	177	11
RLV_6686	Succinate-CoA ligase (ADP-forming) subunit beta	73	47	105	44
RLV_2815	Acetylglutamate kinase	71	50	33	287
DNA replication-, transcription-, and translation-related proteins					
RLV_4236	Elongation factor Tu	288	12	135	22
RLV_6794	50S ribosomal protein L19	159	23	86	59
RLV_4269	50S ribosomal protein L6	119	28	53	142
RLV_4251	Elongation factor G	114	30	44	199
RLV_4538	30S ribosomal protein S2	88	34	84	64
RLV_4246	DNA-directed RNA polymerase subunit beta	85	36	46	182
RLV_7019	DNA gyrase subunit B	74	46	37	251
Other proteins					
RLV_6655	ATP synthase subunit alpha	418	8	111	39
RLV_1848	Glutamyl-tRNAGlu reductase N-terminal domain protein	365	9	128	27
RLV_6653	ATP synthase subunit beta	299	10	297	2
RLV_4905	Protein RecA	232	15	32	308
RLV_6597	Cobaltochelatase CobS subunit	225	17	40	227
RLV_1890	Electron transfer flavodomain protein	222	18	92	50
RLV_1891	Electron transfer flavodomain protein	173	21	56	131
RLV_4578	EF-hand domain pair family protein	106	31	139	19
RLV_4846	FeS assembly ATPase SufC	83	37	124	30
RLV_1886	Glutamyl-tRNAGlu reductase N-terminal domain protein	77	41	70	96
RLV_1961	Nickel-dependent hydrogenase family protein (HupL)	75	43	44	199
RLV_1884	KamA family protein	72	48	113	38

^*a*^ RPSM represents the number of peptide spectra matches (PSMs) normalized to the number of tryptic peptides predicted for the protein.

^*b*^ Rank denotes the position of the proteins related to the full list of proteins identified in RLV_1399 complexes (218 proteins) or in full extracts (1,104 proteins [20]). nd not detected.

The shortlist of the highly abundant proteins coeluting with RLV_1399 includes the structural nitrogenase subunits, which were identified as the most abundant potential interactors of this sHSP. In addition, proteins required for nitrogenase function and biosynthesis (Nif and Fix proteins) were also detected in the analysis but at lower levels. Regarding stress-related proteins, other sHSPs (RLV_6296, RLV_0818, RLV_0502, RLV_2751, and RLV_0817) were identified, suggesting that RLV_1399 might form heterocomplexes with these proteins. RLV_0502 and RLV_0817 were not detected in our previous direct proteomic analysis carried out in pea bacteroids (Table 3; [20]), suggesting that these sHSPs copurified with RLV_1399 are enriched in the eluate fraction. The current analysis has also revealed that RLV_1399 potentially participates in complexes with subunits of ATP-dependent chaperones (GroEL/GroES and DnaK/DnaJ/GrpE) suggesting an involvement of the sHSP in the protein quality control system in bacteroids (33, 36). Also, two universal stress proteins (USPs) have been observed to be highly abundant in the proteomic analysis showing that RLV_1399 might participate in a complex stress-response network. Furthermore, a redoxin family protein, which includes proteins involved in maintaining redox homeostasis and protecting cells against oxidative stress (70), was also identified.

The highly abundant substrate proteins co-eluting with RLV_1399 also included proteins related to carbon and nitrogen metabolism, as a component of the pyruvate dehydrogenase enzyme, and other enzymes as those participating in the tricarboxylic acid cycle (TCA) pathway (succinate-CoA ligase), glyoxylate shunt (succinate semialdehyde dehydrogenase), and extracellular polysaccharides synthesis (UDP-glucose 6-dehydrogenase) (71, 72). In addition, enzymes participating in amino acid synthesis and metabolism have been also observed, such as glutamate synthase or D-3-phosphoglycerate dehydrogenase (73). Furthermore, DNA replication-, transcription-, and translation-related proteins were present in the top list of sHSP-coeluting proteins, including DNA-directed RNA polymerase subunit *β*, DNA gyrase subunit *β*, together with ribosomal proteins and elongation factors Tu and G.

Other prominent potential RLV_1399 substrates included the enzyme glutamyl-tRNA reductase involved in the biosynthesis of delta-aminolevulinic acid, the precursor of tetrapyrrole required for haem groups (74). The potential complex identified might be relevant to keep in a competent state haem proteins as cytochromes, essential for bacteroid respiration, contributing to the survival of the bacteroid as previously suggested (20). Furthermore, we also detected highly prevalent proteins participating in oxidative respiration, such as components of the ATP synthase, and electron transfer flavoproteins likely required for bacteroid respiration under microaerobic conditions. Our analysis also identified one component of the Suf machinery that mediates the iron-sulfur cluster assembly in proteins involved in a number of biological processes, such as electron transfer, redox sensing, and catalysis, playing an important role under oxidative stress and iron starvation limitation (75). Furthermore, HupL, the large structural subunit of the NiFe-hydrogenase, was also detected. Finally, it is interesting to note that, although at position 63, a relevant sHSP-binding partner is the cell division protein FstZ, which is required for the formation of contractile Z-ring prior to the cell division (76).

The data obtained suggest that sHSP RLV_1399 protects a wide diversity of bacteroid proteins against symbiotic stress conditions, being the nitrogenase enzyme one of the most abundant of these potential sHSPs-targets. Given the known role of sHSPs in stabilizing unfolded proteins, we can hypothesize that the “common theme” for this wide range of proteins is their denatured status, likely as a consequence of the different intracellular stresses.

To obtain additional confirmation of the existence of complexes involving the sHSP and NifDKH proteins, desthiobiotin-eluate fractions were further studied by immunoblot analysis using the corresponding antisera. Immunoblot membranes developed with a *Strep-*Tactin-AP conjugated showed the presence of a strong band of the expected size of RLV_1399_ST_ protein that was absent in the eluted fraction from UPM1421(pBBR1MCS-5) included as negative control (Fig. 7B). Similar immunoblots were performed with NifDK antiserum revealing specific bands coeluted with RLV_1399_ST_ of the size corresponding to NifD and NifK proteins that were absent in the eluate from the UPM1421 carrying the empty vector. Immunoblot analysis was also carried out with NifH antiserum. This analysis identified the presence of an immunoreactive band of the size of NifH in the soluble and eluate fractions of bacteroids of the strain expressing RLV_1399_ST_. This band was also present in the soluble fraction of the strain included as negative control but was not observed in the eluate fraction. These data confirm that RLV_1399 participates in complexes with NifDK and NifH proteins.

We were also interested in determining the level of enrichment of these proteins in the eluate fractions. To carry out these studies, we estimated the percentage of the band intensity from the eluate fraction corresponding to RLV_1399_ST_ protein in relation to the total proteins from soluble fraction stained by Coomassie blue (Fig. 7A), obtaining a value of around 50% (Fig. 7C). Further analyses were carried out on the plots generated from the images of the immunoblots. Measurements of plot peak areas relative to the amount of protein subjected to immunoblots analysis resulted in increments of 2.5- and 3.8-fold for NifDK and NifH proteins, respectively, in the eluate fraction in relation to the soluble fraction (Fig. 7D). For RLV_1399_ST_, this value was also estimated, obtaining increments of 10.7-fold in the eluate fractions.

Most of the proteins detected in the RLV-1399-associated protein extract, including nitrogenase components, had been detected previously in the analysis of a bacteroid full extract (20). It is interesting to note that the rank of the identified proteins is quite different in both extracts in a number of cases (Table 3; Table S2). When different, proteins are usually ranked higher in the co-eluted fraction, which is consistent with an RLV_1399-dependent enrichment.

## DISCUSSION

In this work, we study symbiotic sHSPs from *Rlv* UPM791 as molecular chaperones that might prevent protein aggregation and maintain protein homeostasis in response to specific symbiotic stress conditions within the nodule. The phylogenetic analysis of rhizobial sHSPs reveals a probable evolution by gene duplication followed by sequence divergence from an ancient common ancestor as was stated in sHSP evolution studies from different phyla and plants (35, 38).

Our data reveal that the host-dependent sHSP expression is exerted at the transcriptional level. Similar results were obtained with *R. johnstonii* 3841 homologs for RLV_1399 (RL1883) and RLV_6296 (RL4089), both shown to be differentially upregulated in pea and vetch (24) and abundant sHSPs in pea and lentil bacteroids in our system (20). The variable composition of the NCR peptides in IRLC legume nodules (15, 20, 77) suggests that rhizobia might be exposed to different NCR peptides in different hosts as previously reported (8), and this fact could be connected to the host-dependent stress responses in bacteroids. This is also in line with an earlier report in which upregulated genes encoding *E. meliloti* homologs to lentil-specific sHSPs RLV_0817 and RLV_0818 were differentially induced in bacterial cultures exposed to NCR247 and NCR355 peptides (11). In addition, these authors also showed that proteins involved in oxidative stress response (glutathione S-transferase and thioredoxine) were also induced at different levels in an NCR peptide-dependent manner.

Reporter gene analysis has revealed that the *rlv_1399* gene is induced under microaerobic conditions in a FnrN-dependent manner. Previous studies showed that the simultaneous disruption of the two *fnrN* gene copies present in *Rlv* UPM791 suppressed microaerobic induction of *hypBFCDE*, encoding hydrogenase accessory genes, and *fixNOQP*, encoding for *cbb3*-type cytochrome oxidase required for bacteroid respiration (28). The situation is different in the case of *R. johnstonii* 3841, for which FixK controls microaerobic expression in free-living cells and FnrN acts as the main regulator in symbiosis (78). Analysis of the relative mRNA levels of *Rlv* UPM791 *fnrN1* and *fnrN2* genes revealed that these genes are expressed at different levels in the bacteroid, probably due to their location at different replicons as previously stated (21, 28, 29). We have not observed a host effect on gene transcription of *fnrN* genes (data not shown). The presence of two anaeroboxes in the promoter region of *rlv_1399* might be related with the maintenance of precise sHSP levels in the cells, similar to the autoregulation mechanism described for *fnrN1* and *fnrN2* genes and shown to provide appropriate levels of these transcriptional regulators in the cell (29).

To investigate whether anaeroboxes are present in the promoter regions of genes encoding sHSPs in other rhizobial species, a BLAST analysis of the DNA sequence upstream of the *rlv_1399* gene and the 100 nucleotides of their coding sequence was performed. The results identified two Fnr-binding sites in the promoter region of genes encoding to RLV_1399 homologous in other *Rlv* strains (BIHB 1217, RCAM0610, 3841, RCAM2802, and RCAM0626) and *R. johnstonii* 3841 strain. The presence of two anaeroboxes has been also identified in *R. leguminosarum* bv. trifolii (4B, 3B, 23B, 22B, RCAM1365, CC275e, 9B, TA1, and 31B strains), *R. leguminosarum* (Ta6k, Ta9K, Ta6, Ta1k, ATCC14479, and Tp73_4 strains), *Rhizobium* sp. (C104 and TAL182 strains), and *Rhizobium ruizarguesonis* TA1 strain. In *Rhizobium binae* BLR195 strain only, the anaerobox proximal to the translational start site is conserved. The identified genes show a sequence identity of over 90% with RLV_1399 protein. These data suggest that similar regulatory mechanisms of sHSP expression might be conserved in different strains.

It has been previously suggested that FnrN might provide a flexible adaptation to environments in which oxygen concentration changes rapidly (21, 28, 29, 79). This might allow an effective and rapid expression of RLV_1399 sHSP to stabilize the bacteroid proteome under the ultra-low oxygen tensions prevalent in symbiotic conditions. Heat-regulated expression of several sHSP has been described in bradyrhizobia (32, 39), and ROSE elements have been identified in UPM791 lentil-sHSPs, but a similar control of sHSP expression by an Fnr-like protein had not been described so far.

The symbiotically deficient phenotype associated with the *rlv_1399* mutant in pea plants suggests that RLV_1399 might be involved in the adaptation and survival of rhizobia to the symbiotic environment in pea nodules. Previous work revealed that a *R. johnstonii* 3841 mutant in RL1883, the homolog to RLV_1399, was not affected by nitrogen fixation in pea plants (24). The different phenotypes observed might be related to redundancy of functions and a change in the balance of the sHSP in the bacteroids induced by different strains.

Our data are consistent with previous works that showed that the overexpression of *Deinococcus radiodurans* sHSP20 and *E. coli* IbpA/IbpB increases bacterial tolerance to oxidative stress in *E. coli* (80, 81). In addition, a role for sHSP in maintaining redox homeostasis and preventing oxidative enzyme inactivation under these conditions has been also reported (81–83). This is also consistent with the enzymes involved in the response to oxidative stress as potential interactors of RLV_1399 under symbiotic conditions. Regarding the NCR peptides, our results are in line with previous works in which it was observed that the growth of bacterial cultures of *E. meliloti* exposed to *Medicago truncatula* cationic NCR peptides was affected in a concentration-dependent manner (10–12). The antimicrobial efficacy of two *M. truncatula* cationic NCR peptides was previously demonstrated to vary among a wide range of bacteria suggesting either a different mode of action or different sensitivity of proteases to these peptides (11, 13). This might explain the different inhibitory concentrations observed for the cationic NCR peptides in *Rlv* UPM791. The protective role of RLV_1399 might be related to keeping proteins in a soluble state, as antimicrobial activities of NCR peptides have been associated with bacterial targets, including components of the bacterial membranes and a wide range of proteins involved in different cellular functions (3, 8, 10, 12, 13) previously shown to be substrates of bacterial sHSPs (84–88). By contrast, anionic NCR peptides did not show detectable antimicrobial activity (8).

Other sHSPs as potential interactors of RLV_1399 suggest the existence of heterooligomers. Although it has been shown that heterooligomer formation occurs among members of the same class in bacteria (67, 89), some human sHSPs have been reported for their ability to interact with members of both classes (30). Furthermore, the proteomic analysis suggests a potential cooperation involving RLV_1399 in stabilizing denatured proteins and its participation in the cell’s protein quality system, as described for *E. coli* IbpB (33, 83, 88, 90). The observation of universal stress proteins as potential interactors of sHSP indicates that their stabilization might be important in the nodule. These proteins have been reported to facilitate colonization and pathogenicity in bacterial pathogens (91–93) and the role of these proteins in an adaptation to the symbiotic environment has been previously suggested (20). Similar stress responses have been observed under symbiotic conditions and in NCR peptides-treated bacterial cultures. In those cases, upregulated stress-related functions, including those involved in the detoxification of reactive oxygen species, have been identified (11, 23, 24). These responses might operate in our system to face stress conditions required for rhizobia survival in the bacteroid.

Structural nitrogenase subunits (NifKDH) were included among the 50 most abundant in pea-bacteroids (20). A non-specific interaction with RLV_1399 based only on abundance is unlikely, as 17 of such 50 proteins were not detected as potential substrates of RLV_1399. Although the specific targets of rhizobia sHSP have not been previously studied, the NCR247 complex isolated from *M. truncatula* bacteroids induced by *E. meliloti* contained both nitrogenase subunits and GroEL suggesting the existence of an NCR-nitrogenase interaction *via* GroEL (12). In addition, GroEL is required for the nitrogenase structural subunit assembly in other bacteria, and a role in the formation of complexes with other Nif proteins involved in FeMoCo synthesis has also been suggested (94–96).

Multiple proteins involved in a diversity of cellular functions were also previously shown to be targeted and protected from thermal inactivation by sHSPs in other bacteria and yeast (83, 86, 88, 97–99). In addition, cell division protein FtsZ was previously shown to interact with IbpA in other bacteria, and an additional role in assisting FtsZ polymerization contributing to cell division under unfavorable conditions was suggested (100). The observation of this complex in our system is also consistent with the role of NCR247 in cell division inhibition *via* interaction with FtsZ (12).

The diversity of targets that potentially bind to RLV_1399 suggests that this protein might be a general binding protein for denatured substrates of proteins originating under microaerobic and symbiotic conditions. It has to be noted, however, that some of the proteins identified might be indirectly bound to RLV_1399 *via* other proteins that interact directly with the sHSP. Previous reports have stated that the abundance of proteins is not the factor that determines substrate binding to sHSP but it is their tendency to aggregation under stress conditions (83). In addition, hydrophobic sites from sHSPs have been shown to contribute to substrate interaction, in which non-conserved NTE and CTE play the major role, associated with the promiscuity in the substrate recognition by these proteins (30, 34, 36, 101). The independent evolution of NTE and CTE (35) has also been suggested to be responsible for the variations in the substrates interacting with sHSPs from different species (30).

## SUPPLEMENTAL MATERIAL

Figure S1 aem.01385-24-s0001.pdf Sequence alignment of rhizobial sHSPs.

Figure S2 aem.01385-24-s0002.pdf Effect of RLV_1399 expression on the tolerance of Rlv UPM791 to oxidative stress.

Table S1 aem.01385-24-s0003.pdf Peptides and primers used in this work.

Table S2 aem.01385-24-s0004.xlsx Proteins identified in sHSP complexes.

ASM does not own the copyrights to Supplemental Material that may be linked to, or accessed through, an article. The authors have granted ASM a non-exclusive, world-wide license to publish the Supplemental Material files. Please contact the corresponding author directly for reuse.
