# Supplementary material for: A microaerobically induced small heat shock protein contributes to Rhizobium leguminosarum/Pisum sativum symbiosis and interacts with a wide range of bacteroid proteins
Source: Appl Environ Microbiol. 2024 Dec 23;91(1):e01385-24. doi: 10.1128/aem.01385-24 (PMC11784457; doi:10.1128/aem.01385-24)
Supplement: Figure S1 — Sequence alignment of rhizobial sHSPs. [file aem.01385-24-s0001.pdf]

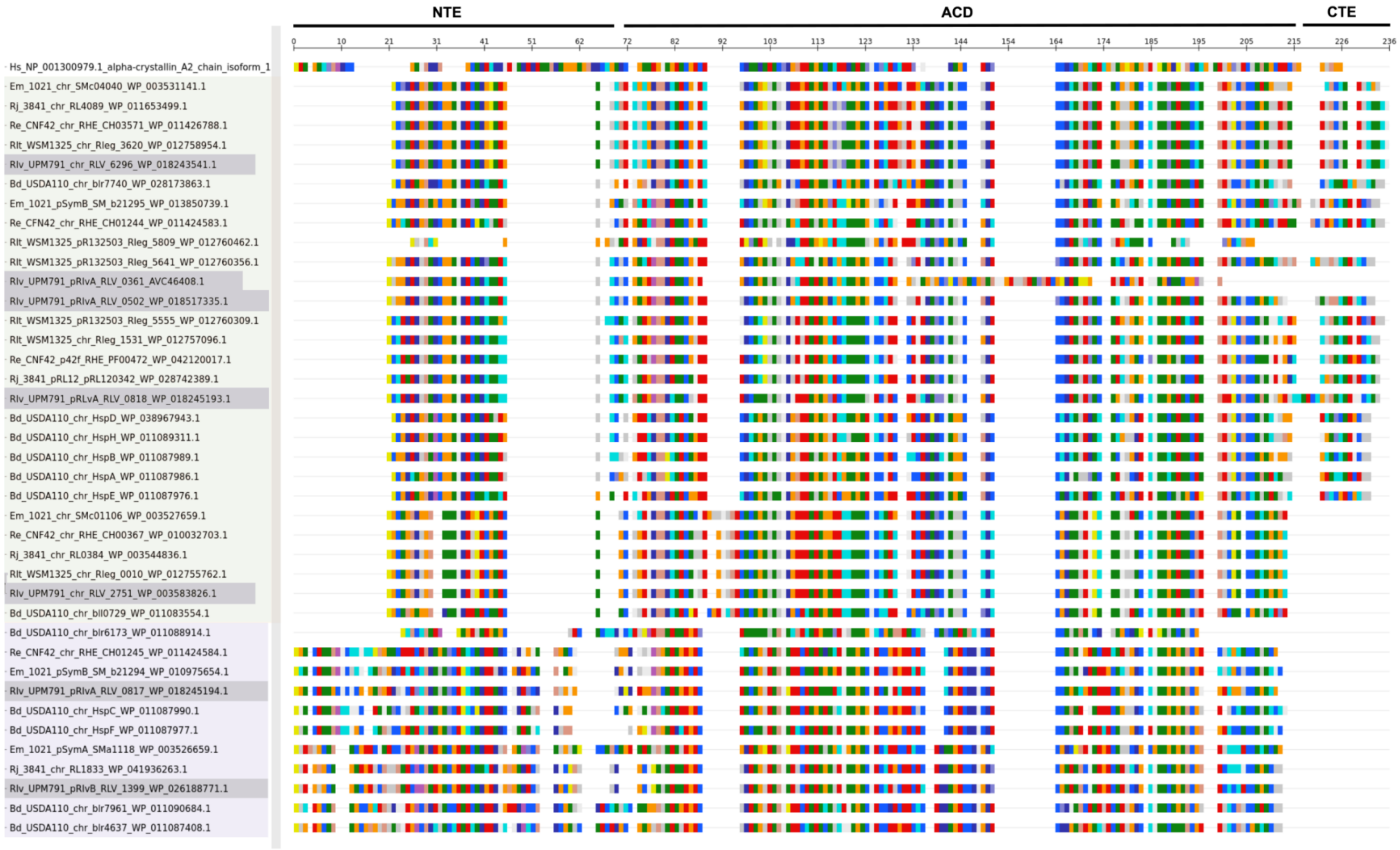
NTE
ACD
CTE
0
10
21
31
41
51
62
72
82
92
103
113
123
133
144
154
164
174
185
195
205
215
226
236
Hs_NP_001300979.1_alpha-crystallin_A2_chain_isoform_1
Em_1021_chr_SMc04040_WP_003531141.1
Rj_3841_chr_RL4089_WP_011653499.1
Re_CNF42_chr_RHE_CH03571_WP_011426788.1
Rlt_WSM1325_chr_Rleg_3620_WP_012758954.1
Rlv_UPM791_chr_RLV_6296_WP_018243541.1
Bd_USDA110_chr_blr7740_WP_028173863.1
Em_1021_pSymB_SM_b21295_WP_013850739.1
Re_CFN42_chr_RHE_CH01244_WP_011424583.1
Rlt_WSM1325_pR132503_Rleg_5809_WP_012760462.1
Rlt_WSM1325_pR132503_Rleg_5641_WP_012760356.1
Rlv_UPM791_pRlvA_RLV_0361_AVC46408.1
Rlv_UPM791_pRlvA_RLV_0502_WP_018517335.1
Rlt_WSM1325_pR132503_Rleg_5555_WP_012760309.1
Rlt_WSM1325_chr_Rleg_1531_WP_012757096.1
Re_CNF42_p42f_RHE_PF00472_WP_042120017.1
Rj_3841_pRL12_pRL120342_WP_028742389.1
Rlv_UPM791_pRLvA_RLV_0818_WP_018245193.1
Bd_USDA110_chr_HspD_WP_038967943.1
Bd_USDA110_chr_HspH_WP_011089311.1
Bd_USDA110_chr_HspB_WP_011087989.1
Bd_USDA110_chr_HspA_WP_011087986.1
Bd_USDA110_chr_HspE_WP_011087976.1
Em_1021_chr_SMc01106_WP_003527659.1
Re_CNF42_chr_RHE_CH00367_WP_010032703.1
Rj_3841_chr_RL0384_WP_003544836.1
Rlt_WSM1325_chr_Rleg_0010_WP_012755762.1
Rlv_UPM791_chr_RLV_2751_WP_003583826.1
Bd_USDA110_chr_bll0729_WP_011083554.1
Bd_USDA110_chr_blr6173_WP_011088914.1
Re_CNF42_chr_RHE_CH01245_WP_011424584.1
Em_1021_pSymB_SM_b21294_WP_010975654.1
Rlv_UPM791_pRlvA_RLV_0817_WP_018245194.1
Bd_USDA110_chr_HspC_WP_011087990.1
Bd_USDA110_chr_HspF_WP_011087977.1
Em_1021_pSymA_SMa1118_WP_003526659.1
Rj_3841_chr_RL1833_WP_041936263.1
Rlv_UPM791_pRlvB_RLV_1399_WP_026188771.1
Bd_USDA110_chr_blr7961_WP_011090684.1
Bd_USDA110_chr_blr4637_WP_011087408.1

FIG S1. Sequence alignment of rhizobial sHSPs. Sequence alignment was created with Clustal Omega. Each color represents different amino acids. *Rlv* UPM791 sHSPs are highlighted in grey. Class A (light green) and Class B (light grey) sHSPs are indicated. Horizontal bars at the top of the figure indicate the extent of the N-terminal (NTE) and C-terminal (CTE) extensions and the α-crystallin domain (ACD). Sequence names shown in the left contain the abbreviate name of the rhizobial species and strains followed by the name of the sHSP protein, indicating whether the localization of the coding sequence is chromosomic (chr) or plasmidic (p), and their accession numbers from GenBank. Bd: *Bradyrhizobium diazoefficiens*; Em: *Ensifer meliloti*; Re: *Rhizobium etli*; Rj: *Rhizobium johnstonii*; Rlp: *R. leguminosarum* bv. phaseoli; Rlt: *R. leguminosarum* bv. trifolii; Rlv: *R. leguminosarum* bv. viciae.
