# Supplementary material for: A microaerobically induced small heat shock protein contributes to Rhizobium leguminosarum/Pisum sativum symbiosis and interacts with a wide range of bacteroid proteins
Source: Appl Environ Microbiol. 2024 Dec 23;91(1):e01385-24. doi: 10.1128/aem.01385-24 (PMC11784457; doi:10.1128/aem.01385-24)
Supplement: Figure S2 — Effect of RLV_1399 expression on the tolerance of Rlv UPM791 to oxidative stress. [file aem.01385-24-s0002.pdf]

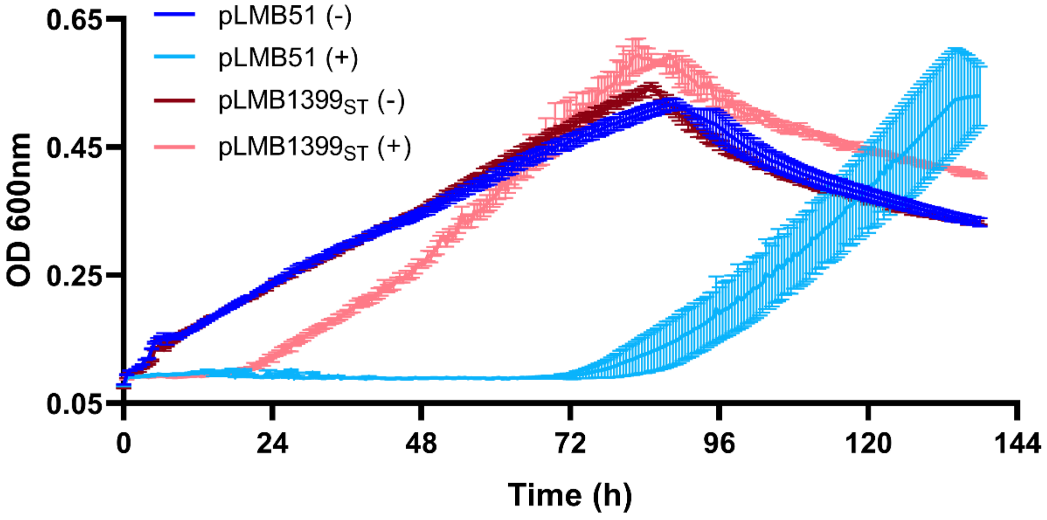

FIG S2 Effect of RLV_1399 expression on the tolerance of *Rlv* UPM791 to oxidative stress. Graph shows the growth of UPM791 derivative strains carrying empty vector pLMB51 or pLMB1399$_{ST}$ plasmid grown under microaerobic (1% O_2) conditions in the absence (-) or presence (+) of 2 mM H_2O_2 in UMS medium. Each OD determination represents the mean of three replicates ± standard error.
