# Supplementary material for: A microaerobically induced small heat shock protein contributes to Rhizobium leguminosarum/Pisum sativum symbiosis and interacts with a wide range of bacteroid proteins
Source: Appl Environ Microbiol. 2024 Dec 23;91(1):e01385-24. doi: 10.1128/aem.01385-24 (PMC11784457; doi:10.1128/aem.01385-24)
Supplement: Table S1 — Peptides and primers used in this work. [file aem.01385-24-s0003.pdf]

Table S1. Peptides and primers used in this work

Peptide	Sequence	pI
G35	LRVKCETDEYCQKVFPSINKSHGVKCI DNLCQFLRKKEVRINT	9.03
G39	VERYKLLPYECEVDEDCPRYIHHPQIM KCINLFCRIVYQD	5.56
L36	SKRNEPVGSFIPCATKLDCPEDMCFPPR RRRCAFNYCECVM	8.11
L40	LDETKYSSKKLFCENSSDCPRSSCYPFS SPICITDICRCI	6.19

Primer	Sequence (5´-3´)	Description
rlv_1399_F	CGAGATCGACCGCGTGTT	*rlv_1399* deletion
rlv_1399 _R	GATCTTCTCGCTGTCCACGC	
PromC_rlv_1399_51_BamHI_F	AAAGGATCCGGCGGCTTTCCTCCTCG ATTGA	Generation of pLMB1399$_{ST}$ and PBBR1399$_{ST}$ plasmids
Prom_rlv_1399_KpnI_F	AAAGGTACCGGCGGCTTTCCTCCTCG ATTGA	
rlv_1399_51_XbaI_Strep_R	AAATCTAGATCACTTTTCGAACTGCG GGTGGCTCCAGCTAGCGGCCGCCTTG ACCGTGATCTTCTTGTCGTT	
rlv_1399_qPCR_F	ATCATTCCACCGAAGCCTGG	qRT-PCR
rlv_1399_qPCR_R	CGCCTTGACCGTGATCTTCT	
rlv_0502_qPCR_F	TATGCACCGCTCTACCGTTC	
rlv_0502_qPCR_R	ATCGAAATCCGGTAGGCGTC	
rlv_0817_qPCR_F	ATGTCTTCCGTGGCTTCGAG	
rlv_0817_qPCR_R	TTTCACCCCGGATGGTCAAG	
rlv_0818_qPCR_F	ACTACCGCATCGTCATTGCT	
rlv_0818_qPCR_R	AGACACCTTGACATGGTCGG	
rpoD_qPCR_F	GCTTCGACCATTTCCTTCTTGG	
rpoD_qPCR_R	GATGAAGTCGATCGGAATCTG	
hupL_qPCR_F	AGAATGGCTACTGGGGCAAC	
hupL_qPCR_R	CCATCGACGTTGATCGGACA	
